# Whole-Genome Analysis of PGP Endophytic *Bacillus subtilis* 10-4: Unraveling Molecular Insights into Plant Growth and Stress Resilience

**DOI:** 10.3390/ijms262411904

**Published:** 2025-12-10

**Authors:** Oksana Lastochkina, Liudmila Pusenkova

**Affiliations:** 1 Institute of Biochemistry and Genetics, Subdivision of the Ufa Federal Research Center of the Russian Academy of Sciences, 450054 Ufa, Russia; 2 Bashkir Research Institute of Agriculture, Subdivision of the Ufa Federal Research Center of the Russian Academy of Sciences, 450059 Ufa, Russia

**Keywords:** *Bacillus subtilis*, endophyte, whole-genome sequencing, plant growth-promoting bacterium, stress resilience, sustainable agriculture

## Abstract

The endophytic bacterium *Bacillus subtilis* 10-4 is a potent bioinoculant, previously shown to enhance growth and resilience to abiotic/biotic stresses across various crops. However, the genetic basis underlying these beneficial traits remains unexplored. In this study, a whole-genome analysis of *B. subtilis* 10-4 was performed to gain the molecular determinants of its plant-beneficial effects. The Illumina MiSeq-based assembly revealed a genome of 4,278,582 bp (43.5% GC content) distributed across 19 contigs, encoding 4314 predicted protein-coding sequences, 42 tRNAs, and 6 rRNAs. This genomic architecture is comparable to other sequenced *B. subtilis* strains. The genomic annotation identified 331 metabolic subsystems with a total number of 1668 functions, predominantly associated with amino acid (281) (16.9%) and carbohydrate (247) (14.9%) metabolism. In silico genomic analysis uncovered a diverse repertoire of genes significant for plant growth and stress resilience. These included genes for colonization (i.e., exopolysaccharide production, biofilm formation, adhesion, motility, and chemotaxis), nutrient acquisition (i.e., nitrogen, phosphorus, iron, potassium, and sulfur metabolisms), and synthesis of bioactive compounds (auxins, salicylic acid, siderophores, gamma-aminobutyric acid, vitamins, and volatiles) and antimicrobials. The latter was supported by identified biosynthetic gene clusters (BGCs) for known antimicrobials (100% similarity) bacilysin, bacillaene, subtilosin A, and bacillibactin, as well as clusters for surfactin (82%), fengycin (80%), and plipastatin (46%), alongside a unique terpene cluster with no known similarity. Additionally, genes conferring abiotic stress tolerance via glutathione metabolism, osmoprotectants (e.g., proline, glycine betaine), detoxification, and general stress response were identified. The genomic evidence was consistent with observed plant growth improvements in laboratory assays (radish, oat) and a field trial (wheat) upon 10-4 inoculation. Thus, the findings elucidate the genomic background of *B. subtilis* 10-4’s beneficial effects, solidifying its potential for utilization as a bioinoculant in sustainable crop production under changing climate accompanied by multiple environmental stresses.

## 1. Introduction

Climate change-induced environmental stresses (drought, extreme temperatures, salinity, etc.) are worsening every year and represent major constraints on agricultural production, threatening global food security [1,2]. Drought intensity in combination with other stresses is projected to increase consistently, which, coupled with the exponential growth of the planet’s population and climate change, only exacerbates the problem and requires urgent solutions to prevent an impending food catastrophe [1,2,3]. Stresses disrupt plant metabolism at the physiological, biochemical, and molecular levels, preventing seed germination and growth, leading to damage to cellular compartments, protein degradation, enzyme inactivation, decreased nutrient absorption, transpiration and photosynthesis, stomatal closure, growth inhibition, wilting, and even death [4,5,6,7,8]. Moreover, drought increases the concentration of chemical compounds introduced into the soil and the occurrence/spread of certain plant diseases, leading to more serious losses in yield and product quality [9,10,11]. Plants are equipped with various defense strategies to counter external threats, but they are not sufficient to protect agricultural crops.

The use of plant–microbe systems, such as beneficial bacterial endophytes, has emerged as a promising approach to address these challenges [12,13,14,15]. *Bacillus subtilis* is one of the most extensively studied plant growth-promoting (PGP) bacteria capable of colonizing the rhizosphere and plants’ internal tissues, providing various benefits to the hosts, enhancing growth and stress resilience under unfavorable biotic (e.g., pathogens [16,17,18], nematodes [19,20], insects [21,22,23]) and abiotic (e.g., drought [24,25,26,27], salinity [28,29,30,31], etc.) conditions. Moreover, *B. subtilis* are generally recognized as safe (GRAS) bacteria [32], which are often considered as microbial factories producing bioactive molecules with a wide range of applications [33]. *B. subtilis*-mediated PGP, antistress, and biocontrol properties are achieved via various mechanisms (i.e., improving nutrient and water availability, altering phytohormone content, photosynthesis, and inducing plants’ systemic resistance), which are summarized in recent reviews [13,34,35], including ours [36,37]. Although the positive effect of *B. subtilis* on the growth and resistance of crops has been widely demonstrated in laboratory conditions, the success of its use in the field is not unambiguous. Moreover, existing studies focus predominantly on single stressors, leaving the effects of combined or multiple stresses poorly understood. Thus, the underlying molecular mechanisms of PGP bacteria, including *B. subtilis*, in plant growth and stress resistance are still not fully clear, which restricts the implementation of microbial-based bioinoculants in large-scale crop production and requires further in-depth investigations.

Our previous extensive research demonstrated that *B. subtilis* strain 10-4 exerts growth-stimulating and antistress actions on various plants, including beans [38], peas [39], potatoes [40], and wheat in laboratory experiments [41,42,43]. This strain also possesses antagonistic activity both in vitro and in planta against phytopathogenic fungi (*Fusarium culmorum*, *F. oxysporum*, *Phytophthora infestans* [44,45], and *Alternaria alternata* [46]), as well as enhanced plant growth under drought [42], salinity [38,39], Cd stress [43], combinations of drought with *F. culmorum* [45,47], and herbicide [48]. The beneficial effects of pre-sowing seed inoculation with *B. subtilis* 10-4 were also revealed under field conditions, demonstrating enhanced growth and yield of crops including sugar beet [46], bean [49], and potato [50]. These features were revealed to be associated with such PGP functional traits as indole-3-acetic acid (IAA) and siderophore production, atmospheric nitrogen (N_2_) fixation [38,41], and surfactin synthesis [47]. Moreover, strain 10-4 successfully colonized plants transferred from seeds (after pre-sowing inoculation) to seedlings (sprouts), thereby further colonizing the entire plant (leaves, stems, roots) as it grows [38,40,41]. Despite the knowledge revealed regarding the physio-biochemical responses of plants to endophytic PGP bacteria *B. subtilis* 10-4 inoculation (e.g., hormonal system [51,52], water balance, photosynthesis [38,48,53], redox-state [54], lignification [38,39]), the genomic insights underlying these impacts on plants remained unexplored. This knowledge gap hinders the reliable and optimized deployment of PGP bacteria, such as the *B. subtilis* 10-4, in real field conditions. Elucidating these intricate plant–microbe mechanisms is therefore crucial for harnessing their full potential to enhance crop production sustainably.

Advancements in high-throughput sequencing technologies have revolutionized microbial genomics, enabling the assembly of complete genomes. These advancements may provide new insights into microbial structure, function, and regulatory networks, establishing a deeper understanding for utilization of endophytic PGP bacteria in agriculture [48,49,50]. Research focused on deciphering the molecular basis of PGP interactions, leveraging genomic tools, is essential. Such understanding will significantly advance the practical application of PGP bacteria, including endophyte *B. subtilis* 10-4, offering robust solutions to pressing global challenges in food security and environmental sustainability.

This study aimed to perform a whole-genome analysis and bioinformatic annotation of the endophytic PGP bacterium *B. subtilis* 10-4 to identify key genetic determinants underlying the biosynthesis of bioactive compounds associated with plant growth and stress resilience and to experimentally validate its efficacy through seed germination assays (radish and oat) and a field (wheat) trial.

## 2. Results

### 2.1. Genome Overview and General Features of B. subtilis 10-4

Whole-genome sequencing of *B. subtilis* strain 10-4 was performed to elucidate its genetic potential for plant growth promotion. The genome sequence was deposited in the DDBJ/ENA/GenBank under the accession JAVHKX000000000, BioProject PRJNA1008864, and BioSample ID SAMN37131992, genome number GCA_035825195.1.

#### 2.1.1. Assembly and Annotation

As a result of genome-wide shotgun sequencing on the Illumina MiSeq platform, 1,032,849 pairs of reads with lengths of 300 (forward) and 115 (reverse) bp were obtained. After filtering, the number of pairs of readings was 1,019,535. Nineteen contigs were obtained; the total length of the assembly is 4,278,582 bp, containing 43.5% G+C. Based on the sequence results, the size of the genome was determined to be 4.3 Mb. During the annotation using prokka v. 1.12 and the genome analysis, in total 4476 sequences (genes), 42 tRNA genes, and 6 rRNA genes were determined (Table 1). Around 4314 protein-coding genes with predicted function were identified.

**Figure 1:**
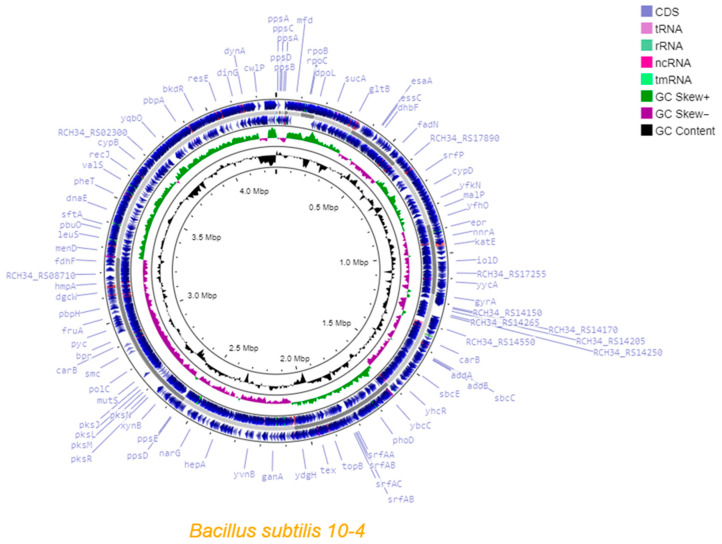
Circular visualization of the genome map of *B. subtilis* strain 10-4. The outermost circle represents the coding sequences of its genome assembly, which was deposited under GenBank BioProject PRJNA1008864 (BioSample ID SAMN37131992).

Circular visualization of the genome map of *B. subtilis* strain 10-4, which involves the distribution of CDS, tRNAs, rRNAs, ncRNAs, tmRNAs, and the GC content skew (constructed using Proksee Server), is presented in Figure 1. The circular genome map reveals several prominent features indicative of its plant-beneficial functions. Most notably, large and dense gene clusters associated with the non-ribosomal synthesis of antimicrobial compounds are clearly visible on the outer ring of the map. These include the plipastatin cluster (e.g., *ppsA-D* genes) at ~0–0.5 Mbp, the surfactin cluster (e.g., *srfAA-AC*) at 1.5–2 Mbp, and the bacillaene cluster (e.g., *pksJ*, *pksL*, *pksM*, *pksN*, *pksR*) at 2.5–3 Mbp. It is important to note that while genes for colonization (e.g., flagellar motility, biofilm formation) and direct growth promotion (e.g., phytohormone, siderophore synthesis, nitrogen metabolism) are present, they are dispersed throughout the genome and are not visualized as distinct clusters on this map. Their detailed annotation and functional assessment are discussed in the following sections based on RAST and antiSMASH analyses.

#### 2.1.2. Taxonomic Assignment and Phylogenetic Position

**Figure 2:**
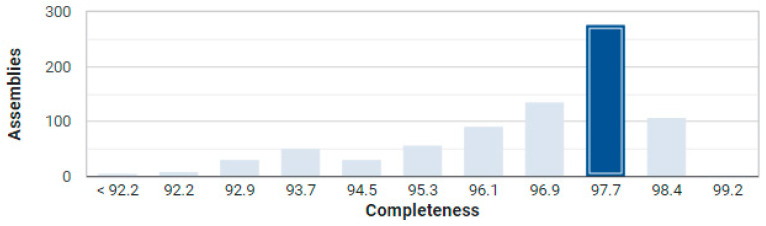
Completeness of *B*. *subtilis* 10-4 with reference sequence (RefSeq) assemblies (dark blue bar). Calculated on the Prokaryotic Genome Annotation Pipeline (PGAP) gene set with the *Bacillus subtilis* CheckM marker set CheckM analysis (v1.2.2).

To confirm the taxonomic assignment, we performed an average nucleotide identity (ANI) analysis. The results showed that strain 10-4 shares the highest ANI with the reference laboratory strain *Bacillus subtilis* subsp. *subtilis* str. 168, with a genome completeness of 97.74% (56th percentile, dark blue bar) and a low contamination of 1.99%, as assessed by CheckM analysis (Figure 2). This firmly places strain 10-4 within the *B. subtilis* species.

**Figure 3:**
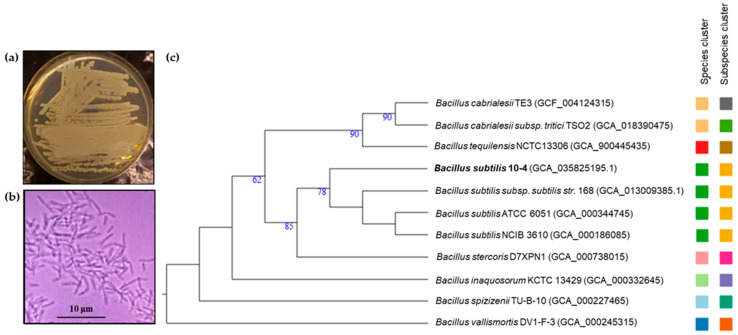
Representative photographs of *B*. *subtilis* 10-4 growth on Luria Bertani (LB) agar medium (24 h at 37 °C) (**a**) and visualization of cells under a Biozero BZ-8100E microscope (Keyence Co., Osaka, Japan) (**b**). Phylogenomic tree BLAST Distance Phylogeny (GBDP) constructed based on whole genome data, showing the position of *B*. *subtilis* 10-4 (**c**). Tree constructed using Type (Strain) Genome Server (TYGS) inferred with FastME 2.1.6.1 from GBDP distances calculated from genome sequences. The branch lengths are scaled in terms of GBDP distance formula d5. The numbers above branches are GBD pseudo-bootstrap support values > 60% from 100 replications, with an average branch support of 80.4%. Different colors on the leaf labels (tips) correspond to identified species and subspecies clusters. The tree was rooted at the midpoint. Genome numbers in GenBank/JGI are given in brackets.

Furthermore, a detailed phylogenomic analysis performed on the Type (Strain) Genome Server (TYGS) image using the Phylogenomic tree BLAST Distance Phylogeny (GBDP) algorithm was automatically selected to compare reference genomes that best match the integrity of the genomic discovery. The resulting tree (Figure 3) shows that strain 10-4 reliably belongs to the *B. subtilis* species and occupies a stable position within it, grouping with neighboring laboratory strain (*Bacillus subtilis* subsp. *subtilis* str. 168) and wild-type strains (*B. subtilis* ATCC 6051 and *B. subtilis* NCIB 3610).

To provide a genomic context for strain 10-4, we compared its general assembly statistics with those of well-known reference strains: the laboratory model strain *B. subtilis* 168 and wild-type plant-beneficial *B. subtilis* strains 26D [16,22], PTA-271 [55,56], Bbv57 [57], and MBB3B9_DBT-NECAB [58] (Table 2). The results showed that the *B. subtilis* 10-4 genome size is within the range of genome sizes of other *B. subtilis* strains exhibiting biocontrol and growth-promoting properties. Some differences are observed in the total number of genes, the number of coding genes, rRNA, tRNA (up to 2-fold differences), and pseudogenes. The number of protein-coding genes in 10-4 is comparable to that of the robust PGP strain Bbv57 and higher than in other PGP strains as well as in the laboratory-domesticated strain 168, suggesting a genetic repertoire that may support diverse functions in natural environments. A detailed functional analysis of specific genes of *B. subtilis* 10-4 contributing to plant growth promotion is presented in the following sections.

### 2.2. Functional Annotation of B. subtilis 10-4 Genome and Identification Plant-Beneficial Genes

**Figure 4:**
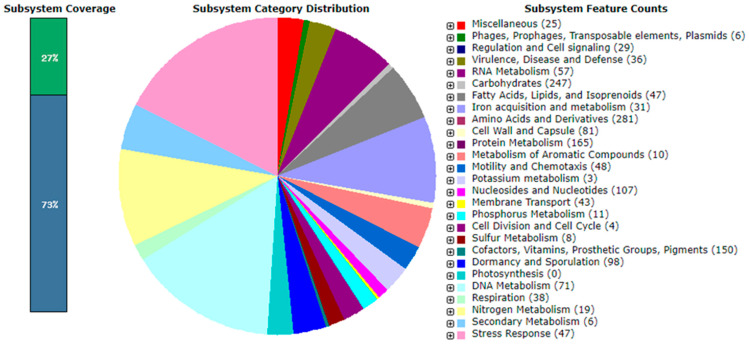
Subsystem analysis of *B. subtilis* 10-4 genome. Distribution of categories of subsystems of cellular metabolism in *B. subtilis* 10-4 based on the results of functional genome annotation using the Rapid Annotation using Subsystem Technology v2.0 (RAST) web service (https://rast.nmpdr.org (accessed on 12 May 2025)). The pie chart represents the percentage of proteins for each category of subsystems. The categories of subsystems are listed in the legend from top to bottom according to the direction of movement on the pie chart clockwise. The numbers in parentheses are the number of metabolic pathways in the corresponding subsystem category. Note: The pie chart displays the 24 most abundant subsystems categories (containing ≥11 features); the legend provides of al 27 categories analyzed by RAST. In subsystem coverage, 27% is indicated with a total of 1196 genes (1136 non-hypothetical and 60 hypothetical) and 73% is not included in subsystem coverage with a total of 3345 genes (1628 non-hypothetical and 1717 hypothetical).

Annotation of the *B. subtilis* 10-4 genome via the Rapid Annotation using Subsystem Technology v2.0 (RAST) web service identified a total of 331 categories of metabolic subsystems—groups of proteins that jointly ensure the implementation of certain biological processes (Figure 4, Table S1). The total number of subsystem functions is 1668. Among the categories of subsystems present in the genome, the subsystems of amino acid metabolism and their derivatives (281) (16.9%), metabolism of carbohydrates (247) (14.9%), and proteins (165) (9.9%) were the most represented. The subsystems of cofactors, vitamins, prosthetic groups, pigments (150) (8.88%), nucleosides and nucleotides (107) (6.34%), dormancy and sporulation (98) (5.8%), cell wall and capsule (81) (4.79%), RNA metabolism (57) (3.37%), fatty acids, lipids, and isoprenoids (47) (2.78%), DNA metabolism (71) (4.2%), motility and chemotaxis (48) (2.84%), stress response (47) (2.78%), membrane transport (43) (2.54%), respiration (38) (2.25%), virulence, disease, and defense (36) (2.13%), iron acquisition and metabolism (31) (1.83%), regulation and cell signaling (29) (1.71%), miscellaneous (25) (1.48%), nitrogen metabolism (19) (1.13%), phosphorus metabolism (11) (0.65%), metabolism of aromatic compounds (10) (0.59%), sulfur metabolism (8) (0.47%), secondary metabolism (6) (0.35%), phages, prophages, transposable elements, plasmids (6) (0.35%), cell division and cell cycle (4) (0.24%), and potassium metabolism (3) (0.17%) were also identified. Furthermore, in subsystem coverage, 27% is indicated with a total of 1196 genes (1136 non-hypothetical and 60 hypothetical), and 73% is not included in subsystem coverage, with a total of 3345 genes (1628 non-hypothetical and 1717 hypothetical).

*Amino acids and derivatives*. The genomic analysis of *B. subtilis* 10-4 revealed an extensive and diverse repertoire of genes dedicated to the metabolism of amino acids and their derivatives, comprising 281 subsystem categories (Figure 4). This highlights a high metabolic plasticity for nitrogen assimilation and amino acid biosynthesis. The largest functional group within this subsystem was dedicated to glutamine, glutamate, aspartate, asparagine, and ammonia assimilation (36 categories). Key annotated genes in this central nitrogen metabolic network include *PgsA*, *PgsB*, *PgsC*, *PgsE*, *PgsS* (involved in poly-gamma-glutamate synthesis), *GltB*, *GltD* (components of glutamate synthase), *AspA* (aspartate ammonia-lyase), *AspC* (aspartate aminotransferase), *AsnB*, *AnsA*, *AnsB* (asparagine synthetases/asparaginases), and *GlsA* (glutaminase). Furthermore, the genome encodes comprehensive pathways for biosynthesis of all essential amino acid families: (i) Aromatic amino acids (38 categories), featuring genes for the shikimate pathway (*AroB*, *AroC*, *AroD*, *AroE*, *AroG*, *AroH*, *AroJ*) and specific branches for tryptophan (*TrpA*, *TrpB*, *TrpC*, *TrpD*, *TrpE*), tyrosine (*TyrA*), and phenylalanine (*PheA*); (ii) Branched chain amino acids (40 categories), including *IlvA, IlvD*, *IlvE*, *IlvG*, and *IlvM* for valine and isoleucine biosynthesis, and *LeuA*, *LeuB*, *LeuC*, *LeuD* for leucine synthesis; (iii) Lysine, threonine, methionine, and cysteine (81 categories), comprising genes such as *Hom* (homoserine dehydrogenase), *ThrA*, *ThrB*, *ThrC* (threonine biosynthesis), *MetE*, *MetP*, *MetN*, *MetQ* (methionine acquisition), *DapH* (lysine biosynthesis), and *CysE* (cysteine synthesis); (iv) Arginine, urea cycle, polyamine, and gamma-aminobutyric acid (GABA) metabolism (30 categories), with genes including *ArgF*, *ArgR* (arginine metabolism), *RocA*, *RocB*, *RocD*, *RocE*, *RocF*, *RocR* (arginine catabolism), *SpeA*, *SpeB* (polyamine synthesis), and *UreA*, *UreB*, *UreC* (urea hydrolysis). Notably, we identified *gabaT* (4-aminobutyrate aminotransferase), a key gene for the catabolism of GABA. This suggests that strain 10-4 can utilize this important plant signaling molecule as a nutrient source, potentially enhancing its fitness in the rhizosphere. Additionally, other essential pathways were identified, including histidine metabolism (13 categories, e.g., *HisB*, *HisC*, *HisD*, *HisF*, *HisG*, *HisH*, *HisI*, *HutG*, *HutH*, *HutI*, *HutU*), proline biosynthesis (11 categories, *proA*, *proB*, *proC*, *proG*), and metabolism of alanine, serine, and glycine (32 categories, e.g., *SerA*, *SerC*, *SerC*, *SerS*, *GlyA*, *CysA*, *CysB*). This genetic capacity for comprehensive amino acid biosynthesis, interconversion, and utilization of key plant-derived compounds like GABA underscores the metabolic autonomy and ecological adaptability of strain 10-4. The presence of these pathways represents a key genomic adaptation for competing successfully in the rhizosphere environment, where the ability to synthesize essential metabolites and exploit available nutrients is critical for survival and plant colonization.

*Carbohydrates.* Genomic analysis of *B. subtilis* 10-4 uncovered a broad metabolic capacity for carbohydrate utilization, encompassing 247 subsystem categories (Figure 4). This extensive repertoire indicates a strong potential for efficient energy generation and carbon sourcing from diverse substrates. The most prominent group within this subsystem was dedicated to central carbohydrate metabolism (79 categories), including genes such as *pdhA* (a component of the pyruvate dehydrogenase complex), which is crucial for linking glycolysis to the tricarboxylic acid (TCA) cycle. A key feature of the strain’s genetic arsenal is its capacity to utilize a wide range of monosaccharides and simple sugars (74 categories). This includes pathways for the uptake and catabolism of pentoses (e.g., *XylA*, *XylB*, *AraA*, *AraB*), hexoses (e.g., *FruK)*, and various uronic acids (e.g., *UxuA*, *UxuB*, *KduI*), suggesting an ability to degrade and ferment multiple plant-derived carbohydrates. Furthermore, the genome encodes specialized pathways for the metabolism of other carbohydrate classes: (i) Di- and oligosaccharides (10 categories, e.g., *GalK*, *GalE, SucP*), allowing for the use of sucrose, galactose, and related compounds; (ii) Aminosugars (9 categories, e.g., *NagA*, *NagB*), which are key components of bacterial cell walls, indicating recycling capabilities; (iii) Sugar alcohols (20 categories, e.g., *GlpK*, *GlpD* for glycerol; the lol operon for *myo*-inositol), providing metabolic flexibility under different osmotic conditions; (iv) Fermentation (26 categories), with genes for mixed-acid fermentation (e.g., *pta*, *atoB*) and butanoate metabolism (e.g., *bdhA*, *HbdA*). Critically, we identified the transcriptional regulator *alsR*, which controls the acetoin biosynthesis pathway. This suggests the strain’s potential to produce acetoin, a neutral metabolite important for pH homeostasis and plant interactions; (v) Polysaccharides (5 categories, with genes annotated as *GAT_C*, *GAT_D*, *GBr*, *GS*, *GP*), indicating the strain’s genetic potential for the production of extracellular polysaccharides (EPS); (vi) Organic acids (17 categories, e.g., *PrpB*, *PrpC)* and one-carbon metabolism (5 categories, e.g., *folD*, *MTFR)* were also identified, rounding out the strain’s core metabolic networks. The genetic blueprint of strain 10-4 reveals a “generalist” strategy for carbon utilization. The presence of numerous and diverse pathways for sugars, sugar alcohols, and organic acids is a key genomic adaptation for survival in the competitive and nutrient-heterogeneous rhizosphere. Furthermore, the capacity for EPS synthesis is a critical trait for biofilm formation, which facilitates root colonization and protects against environmental stresses. This metabolic versatility enables the strain to efficiently capitalize on the root exudates of a variety of host plants, supporting its establishment and proliferation as a plant-beneficial bacterium.

*Protein metabolism.* Analysis of strain 10-4 genome subsystems associated with protein metabolism revealed its complex nature (Figure 4). The largest group consisted of genes responsible for protein biosynthesis (123 categories), which reflects the strain’s high metabolic capacity for growth and reproduction. Key components of the translation apparatus were identified, including initiation (e.g., *IF-1*, *IF-2*, *IF-3*), elongation (e.g., *EF-Tu*, *EF-G*), and termination (e.g., *RF-2*) factors, as well as genes encoding numerous ribosomal proteins. Furthermore, systems that ensure protein quality control in the cell were also thoroughly annotated. This includes molecular chaperones (such as *DnaK*, *DnaJ*, and *GrpE*) and enzymes for protein folding (8 categories, including *PrsA*, which is crucial for the secretion of extracellular proteins in *Bacillus*). Additionally, the genome contained a diverse and extensive set of genes for protein degradation (24 categories), including ATP-dependent proteolytic complexes (e.g., *ClpC*, *ClpX*, and *LonI*) essential for recycling damaged proteins and regulating cellular processes, as well as enzymes for processing peptides and amino acids. The presence of these systems for protein turnover and amino acid recycling is likely a key genomic adaptation for survival of the strain in the competitive and nutrient-variable rhizosphere environment.

*Membrane transport.* It identified 43 subsystem categories responsible for membrane transport (Figure 4), revealing a sophisticated apparatus for nutrient uptake, ion homeostasis, and protein secretion. The most diverse group within this subsystem consisted of Energy-Coupling Factor (ECF) class transporters (11 categories). These include four essential cofactors and vitamins such as thiamine (*ThiT*), riboflavin (*RibU*), biotin (*BioY*), and tryptophan (*TrpP*), highlighting the strain’s capability for efficient scavenging of vital nutrients from the environment. A significant portion of the transport systems was dedicated to cation transporters (13 categories). Notably, the genome encodes a comprehensive copper transport system (*CopA*, *CopC*, *CopD*, *CopZ*, and the regulator *CsoR*), which is crucial for detoxification and survival in environments with fluctuating copper levels. Additionally, genes for magnesium transport (*corA*) and sodium-dependent phosphate transporters (*nptA*) were identified, underscoring the strain’s ability to maintain ionic balance. The strain 10-4 also possesses systems for the transport of specific metabolites, including a tripartite ATP-independent periplasmic (TRAP) transporter (*DctP*) for C4-dicarboxylates and an ATP-binding cassette (ABC) transporter for oligopeptides (*oppA*), facilitating the uptake of peptides as a nutrient source. Furthermore, the genome encodes essential machinery for protein translocation across the cytoplasmic membrane (9 categories). This includes the twin-arginine translocation (Tat) system (*TatAd*, *TatCd*), which secretes folded proteins, and the exoprotein secretion system EcsAB transporter (*EcsA*, *EcsB*, *EcsC*), which affects the expression and secretion of extracellular proteins. The diverse membrane transport systems identified in the genome of strain 10-4 are integral to its success as a rhizosphere colonizer. The capacity for high-affinity uptake of vitamins, peptides, and ions, coupled with robust metal detoxification and protein secretion pathways, provides a strong competitive advantage in the nutrient-limited and potentially stressful plant root environment.

*Nucleosides and nucleotides.* Subsystem analysis revealed an extensive genetic repertoire for nucleoside and nucleotide metabolism in strain 10-4, comprising 107 categories (Figure 4), which underscores a robust capacity for nucleic acid biosynthesis, salvage, and recycling. The genome encodes a nearly complete pathway for de novo purine biosynthesis (54 categories), featuring key genes such as *PurF*, *PurD*, *PurL*, *PurM*, and *PurC.* This is complemented by a sophisticated system for purine conversion and salvage, including genes for xanthine dehydrogenase *(XdhC*, *XDHFeS*, *XDHMo*) and nucleoside interconversions (*GMPR*, *guk*, *ndk*), enabling the strain to efficiently utilize exogenous purines. Similarly, a comprehensive pathway for pyrimidine metabolism (35 categories) was identified. This includes the complete de novo synthesis pathway (*pyrAA*, *pyrAB*, *pyrC*, *pyrF)* alongside a versatile set of enzymes for pyrimidine conversions (*ctps*, *udk*, *cytk*, *thyk*), allowing for the synthesis and interconversion of all pyrimidine nucleotides. Notably, the genome also possesses specialized systems for detoxification (4 categories), including genes such as *MazG* (nucleoside triphosphate pyrophosphohydrolase) and *NudF* (ADP-ribose pyrophosphatase). These enzymes are crucial for maintaining cellular nucleotide pools and mitigating the toxicity of abnormal nucleotides under stress conditions. The genetic complement for nucleotide metabolism in strain 10-4 provides a high degree of metabolic autonomy and flexibility. The capacity for both de novo synthesis and efficient salvage of purines and pyrimidines ensures the availability of essential precursors for DNA and RNA synthesis during rapid growth and in nutrient-fluctuating environments like the rhizosphere. Furthermore, the presence of dedicated detoxification systems highlights a sophisticated mechanism for managing nucleotide-related metabolic stress, contributing to the overall resilience of the strain.

*RNA metabolism.* Subsystem analysis identified 57 categories associated with RNA metabolism in strain 10-4 (Figure 4), reflecting a comprehensive machinery for genetic regulation and RNA homeostasis. The largest functional group was dedicated to transcription (34 categories). The genome encodes the core RNA polymerase subunits (alpha, beta, delta, and omega) and a diverse repertoire of sigma factors, including the primary sigma factors (*RpoD*) and alternative factors involved in flagellar biosynthesis (*FliA*), sporulation (*SigF*, *SigG*), heat shock (*SigH*), cell envelope stress (*SigW*), and other specific regulatory roles (*SigZ*, *SigV*, *SigE*, *SigI*, *RpoN*). This diversity enables precise promoter recognition and rapid reprogramming of gene expression in response to environmental changes. Furthermore, key transcription factors and regulators were identified, including elongation factors (*NusA, NusG*), termination factor (*Rho*), and regulators of the Rrf2 family (*IscR*, *NsrR*), which are critical for managing transcriptional fidelity and responding to specific stimuli. The genome also possesses an extensive suite for RNA processing and modification (23 categories). This includes various ribonucleases responsible for RNA degradation and maturation, such as *RNase II*, *RNase R* (for mRNA turnover), *RNaseHII/HIII* (for processing RNA-DNA hybrids), and *RnhB*/*C* (ribonucleases H). Additionally, enzymes for tRNA and rRNA modification were annotated, including *RsmA/B/D* (for rRNA methylation) and *TrmL* (tRNA methylation), which are essential for fine-tuning translation efficiency and fidelity. The elaborate systems for transcription regulation and RNA processing equip strain 10-4 with a high degree of transcriptional plasticity and post-transcriptional control. This genetic foundation is pivotal for adapting its metabolic and physiological states swiftly, a critical advantage for competing and thriving in the dynamic and often stressful rhizosphere environment.

*DNA metabolism.* It was revealed 71 subsystem categories for DNA metabolism (Figure 4), which underscores a robust capacity for genomic maintenance, replication, and genetic plasticity. The core of this system is a sophisticated apparatus for DNA repair (50 categories), significantly outnumbering other groups and highlighting the strain’s emphasis on genomic integrity. This includes multiple specialized pathways: (i) Base Excision Repair (BER) systems (e.g., *MutY*, *EndoIV*) for correcting base damage; (ii) Nucleotide Excision Repair (NER) machinery (*UvrA*, *UvrB*, *UvrC*) for fixing bulky DNA lesions; (iii) Mismatch Repair (MMR) components (*MutS, MutL*) to ensure replication fidelity; and (iv) Recombinational repair systems, centered around the key recombinase *RecA* and its regulators (*RecF*, *RecO*, *RecR*, *RecX*), which are essential for restarting stalled replication forks and repairing double-strand breaks. While the DNA replication machinery (6 categories) was annotated with core enzymes like the DNA polymerase III subunit *PolC* and topoisomerase *GyrA*, the extensive repair systems suggest a primary genetic investment in correcting DNA damage rather than merely duplicating the genome. The exceptional investment in diverse DNA repair pathways equips strain 10-4 with a high degree of genomic resilience. This is a critical adaptive trait for survival in the rhizosphere, where bacteria are exposed to a range of DNA-damaging agents, including UV radiation, reactive oxygen species, and metabolic toxins from competing microbes. The concurrent presence of competence and restriction systems further illustrates a strategic balance between acquiring new genetic traits and protecting genomic integrity.

#### 2.2.1. Genes Involved in Colonization and Interactions with Plants

*Exopolysaccharide (EPS) production and biofilm formation*. The ability of *B. subtilis* 10-4 to effectively colonize the surface of roots and internal tissues of plants is a prerequisite for phytostimulation. The results showed that the genome of 10-4 contains a complete set of genes for biofilm formation, including the major exopolysaccharide (EPS) (alginate) operon epsA-O (*epsO*, *epsN*, *epsM*, *epsL*, *epsK*, *epsJ*, *epsI*, *epsH*, *epsG*, *epsF*, *epsE*, *epsD*), essential for producing the biofilm matrix, and genes for a pectin-like polysaccharide (PEL) (*pel*, *pelB*), which contributes to structural integrity and adhesion. This capacity for robust biofilm formation, mediated by the *eps* and *pel* genes, is a key factor enabling strain 10-4 to anchor itself firmly to plant surfaces and form protective microcolonies. However, successful colonization is a complex process dependent on many other genes encoding adhesion factors (flagella, pili), secretion systems, metabolic pathways, and plant defense evasion mechanisms that provide a competitive advantage in the rhizosphere niche.

*Bacterial chemotaxis, motility, and colonization*. Genomic analysis confirmed that *B. subtilis* 10-4 possesses an extensive genetic system for flagellar assembly and motility, a critical trait for root surface colonization. A total of 48 subsystem categories were identified, forming a near-complete pathway for a functional flagellum (Figure 4). It was revealed all essential components for a motile phenotype, including (i) Structural core and assembly apparatus, such as genes encoding the basal body (*FliF*, *FlgB*), the hook (*FlgE*), the filament (*FlaA*), and the torque-generating MotAB motor complex; (ii) Regulatory network, i.e., key transcriptional regulators, including the sigma factors RpoD and RpoN, and specific regulators (*FlbD*, *FlhF*) that control the expression and assembly of flagellar genes; and (iii) Assembly control and chaperones, i.e., factors ensuring proper construction, such as the hook-length control protein FliK and chaperones (*FliS*, *FliT*, *FlgN*) that prevent premature aggregation of filament subunits. While subsystems and genes encoding social motility and magnetotaxis were not detected using the RAST server, the presence of the *PomA* gene, associated with chemotaxis in some systems, suggests retained sensory capability. The comprehensive nature of the flagellar biosynthetic machinery in strain 10-4 provides a strong genomic basis for active swimming towards plant roots, a crucial first step in establishing a successful plant-bacteria interaction and effective root colonization.

#### 2.2.2. Metabolic Pathways of *B. subtilis* 10-4 Underlying PGP Capabilities and Stress Resistance

##### Functional Genes Associated with Plant Growth and Mineral Nutrition

The analysis of *B. subtilis* 10-4’s genome showed the presence of several genes associated with plant growth promotion and mineral nutrition enhancement, such as the production of auxin, the metabolism of nitrogen, phosphorus, potassium, and sulfur, as well as iron acquisition and metabolism (Figure 4, Table 3).

*IAA synthesis.* The genomic analysis indicates a strong potential for the biosynthesis of the phytohormone auxin (indole-3-acetic acid, IAA) in strain 10-4. Four subsystems dedicated to this pathway were identified (Figure 4). Crucially, the genome encodes a nearly complete pathway for the synthesis of tryptophan, the primary precursor for IAA. This includes key enzymes such as anthranilate synthase (*trpE*), tryptophan synthase (*trpA*, *trpB)*, indole-3-glycerol phosphate synthase (*trpC*), putative tryptophan transport protein *(trpP*), anthranilate phosphoribosyltransferase *(trpD*), N-(5′-phosphoribosyl) anthranilate isomerase *(trpF*), and tryptophan-tRNA ligase (*trpS*), which are involved in the synthesis of IAA precursor tryptophan. The presence of this integrated genetic system—from tryptophan biosynthesis to its potential transport—suggests that strain 10-4 is genetically equipped to synthesize and possibly deliver IAA precursors, a key mechanism for directly stimulating plant root development and facilitating its own establishment in the rhizosphere.

*Nitrogen metabolism.* It was found that nitrogen metabolism in the strain 10-4 genome is supported by 19 subsystems, encompassing key processes for nitrogen utilization and adaptation (Figure 4, Table 3). This includes six subsystems for denitrification (involving genes such as *NarG*, *NarH*, *NarJ*, *NarI*, *NorD*, and *NorQ*) and thirteen for ammonia assimilation, nitrate/nitrite ammonification (e.g., *NarG*, *NarH*, *NarJ*, *NiR1a*, and *NiR1b*), and the response to nitrosative stress (regulated by *NsrR*). Further analysis confirmed the presence of core genes essential for nitrogen regulation and metabolism. These include the global nitrogen regulator *glnG* and the PII protein *nrgB*, which coordinate the cellular response to nitrogen availability. The genome also encodes pathways for ammonium incorporation via glutamine synthetase (*glnA)* and glutamate synthase (*gltA*, *gltB*), as well as systems for nitrate transport (*narT*, *nasA*) and its assimilatory reduction to ammonia *(nasB*, *nasC*, *nasD*, *nasE*).

*Phosphorus, potassium, sulfur, and iron metabolism*. Genomic analysis confirmed that *B. subtilis* 10-4 possesses a comprehensive suite of genes for the metabolism of key inorganic nutrients, facilitating its adaptation to diverse environments. The strain’s genome encodes 31 subsystems dedicated to iron acquisition and metabolism (Figure 4, Table 3). A significant portion of these (15 subsystems) is specialized for the synthesis and uptake of siderophores, as well as the utilization of heme and hemin, which are crucial for scavenging iron under limiting conditions. Additionally, the genome contains the *EfeUOB* operon, encoding a ferrous iron transporter induced at low pH, providing an alternative strategy for iron uptake in acidic environments like the rhizosphere. For other essential elements, the analysis revealed 11 subsystems for phosphorus metabolism, 8 for sulfur metabolism, and 3 for potassium homeostasis. Specifically for phosphate acquisition, key genes for both high-affinity ABC transport (*pstS*, *pstA*, *pstB*, *pstC*) and low-affinity transport (*pitA*) were identified, ensuring phosphate scavenging across a range of environmental concentrations.

##### Functional Genes Associated with Stress Tolerance

The results showed that *B. subtilis* 10-4 possesses a robust genetic arsenal for stress tolerance, comprising 47 subsystem categories (Figure 4, Table 4). These systems equip the strain to survive in challenging environments. The stress response network includes dedicated mechanisms for: (i) Oxidative stress (14 categories), featuring key genes for reactive oxygen species (ROS) detoxification, including superoxide dismutase (*sodB*, *sodC*) and the peroxidase *AhpC*, alongside regulators like *PerR*; (ii) Osmotic stress (15 categories), centered around compatible solute synthesis and uptake, such as the *opu* and betaine gene clusters (*OpuAA*, *OpuAC*, *BetB*, etc.), which are crucial for maintaining cellular turgor under drought or salinity; (iii) General stress response, regulated by the alternative sigma factor SigB and its associated regulators (*RsbR*, *RsbS*, *RsbT*, *RsbU*, *RsbV*, *RsbW*). This system orchestrates a broad defense program against various environmental insults. Additional systems for carbon starvation (*CstA*, *CsrA*) and periplasmic stress (*RseP*) further underscore the strain’s preparedness for nutrient limitation and protein folding challenges. The presence of this multifaceted stress tolerance machinery, including general stress proteins (*ysnF*, *yhdN*), is a key genomic determinant of the strain’s resilience. It provides a competitive advantage for survival and persistence in the fluctuating and often stressful conditions of the plant root environment.

*Cell signaling*. It was identified 29 subsystem categories responsible for cell signaling and regulatory functions in *B. subtilis* 10-4 (Figure 4), revealing sophisticated systems for environmental sensing and cellular response coordination. The strain possesses comprehensive machinery for carbon catabolite repression and nutrient sensing (14 categories), including the core components of the HPr system (*CcpA*, *PtsH*, *Crh*) and regulators of (p)ppGpp signaling (*SpoT*, *Spo*), which enables precise metabolic prioritization in response to nutrient availability. Furthermore, the genome encodes multiple programmed cell death and toxin-antitoxin systems (15 categories), featuring key genes such as *cidA*, *cidB*, *lrgA*, *lrgB*, and *lytS*. These systems regulate bacterial altruism, biofilm development, and stress survival through controlled cell lysis and growth regulation, representing an important adaptation for population-level fitness in competitive environments. The integration of these regulatory networks provides strain 10-4 with the capability to dynamically coordinate metabolism, growth, and survival strategies in response to environmental fluctuations, enhancing its ecological fitness.

*Other unique genome scaffolds of strain 10-4 relevant to stress tolerance.* Genomic analysis of *B. subtilis* 10-4 uncovered several unique genetic determinants that contribute to its stress resilience and plant interaction capabilities. Notably, the genome contains the *pchA* gene, encoding a salicylate biosynthesis isochorismate synthase, indicating the potential for salicylic acid (SA) production, a phytohormone known to induce systemic resistance in plants. The strain’s arsenal for metabolic stress management includes genes for the synthesis of pyridoxal 5′-phosphate (vitamin B6) (*pdxT*, *pdxS*), a potent antioxidant, and enzymes for gamma aminobutyric acid (GABA) metabolism (*gabD*) and polyamine synthesis (*speE*), which are crucial for maintaining cellular homeostasis under various abiotic stresses. Furthermore, the genome encodes molecular chaperones (e.g., *hslR*) for protein protection during heat shock, and key genes for the production of volatile organic compounds (VOCs) such as acetoin (*alsS*, *alsD*) and butanediol (*bdhA*), which can enhance plant stress tolerance and facilitate root colonization. The co-occurrence of these specialized genetic features—spanning phytohormone potential, antioxidant synthesis, stress metabolite production, and protective volatiles—provides strain 10-4 with a multifaceted and robust mechanism to mitigate environmental stresses, thereby supporting its survival and plant-beneficial functions.

##### Secondary Metabolite Biosynthetic Genes in *B. subtilis* 10-4 Genome

The genome of *B. subtilis* 10-4 encodes a suite of enzymes and antimicrobial compounds indicative of a strong potential for biocontrol and host interaction (Figure 4). This includes genes for cell wall-degrading enzymes such as β-glucanase (*bglS*) and endoglucanase (*eglS*), which can lyse fungal pathogens. Furthermore, a diverse array of proteases (e.g., *clpX*, *lon, wprA*, *aprX*) was identified, which are involved in nutrient acquisition, protein turnover, and possibly the degradation of pathogenic fungal proteins. Crucially, the genome contains the complete surfactin biosynthesis surfactin operon (*srfAA-AD*), responsible for producing this potent lipopeptide with well-documented antifungal, antibacterial, and surfactant properties. The co-occurrence of these lytic enzymes and the potent biosurfactant surfactin provides a multi-faceted molecular arsenal, positioning strain 10-4 as a promising candidate for the biocontrol of plant pathogens and for facilitating root colonization.

The results of analysis using antibiotics and secondary metabolite analysis shell (antiSMASH) web service confirmed the presence in the genome of *B. subtilis* 10-4 biosynthetic gene clusters (BGCs) involved in the synthesis of various secondary metabolites, including T3PKS (III polyketide synthases), NRPS (nonribosomal peptide synthetases), glycine-rich peptides (betalactones, rantipeptides, sactipeptides), non-ribosomal (NP)-metallophones, transAT-PKS (trans acyltransferase polyketide synthetases), and terpenes (Table S2). Particularly, the gene clusters associated with the synthesis of bacillibactin (*dhbA*, *dhbB*, *dhbC*, *dhbE*, *dhbF*, *pncA*, *bznD*, *ald*, *mbtH*, *besA*, *yumB*, *yumC*, *yutJ*), bacillaene (*pksB*, *pksD*, *pksS*, *pksR*, *pksH*, *pksI*, *pksN*, *pksM*, *pksL*, *pksJ*, *pksG*, *pksF*, *fabD*, *aprX*, *acpK*, *miaB*, *kbl*, *tdh*, *pbpX*), bacilysin (*bacD*, *bacC*, *bacG*, *bacF*, *ywhC*, *rocC*, *pruA*, *spsL*, *spsI*, *spsG*, *spsC*, *rfbD*, *rfbB*), subtilosin A (*sboA*, *narG*, *albA*, *albE*, *albF*), surfactin (*srfAA*, *srfAB*, *srfAC*, *srfAD*, *nasB*, *zinU*, *yckB*, *ycxD*, *tcyA*, *ubiX*, *yclE*), fengicin (*ppsD*, *ppsE*, *ldeI*, *ldeJ*, *ldeHA*, *ldeF*, *ldeE*, *galU*), plipastatin (*ppsA*, *ppsC*), terpenes (*sqhC*, *dhaS*), and sactipeptide and ranthipeptide (*skfA*, *skfB*, *skfC*, *ybdG)* were detected in the *B. subtilis* 10-4 genome. Among these, five clusters exhibited 100% similarity to known pathways for bacillaene, subtilosin A, bacilysin, bacillibactin, and sporulation-killing factor, confirming the presence of this conserved core set of secondary metabolites. The clusters for the key lipopeptides surfactin and fengycin showed 82% and 80% similarity to their known counterparts, respectively, suggesting potential structural or regulatory variations. The similarity of other BGCs ranged from 46% for plipastatin to 13% for 1-carbapen-2-em-3-carboxylic acid, indicating a spectrum of divergence. Most notably, the analysis uncovered a unique and potentially novel terpene BGC in region 9 with no similarity to any known clusters in the database, indicating a high probability of novelty (Table S1). The presence of this multi-faceted metabolic arsenal—spanning siderophores, broad-spectrum antibiotics, and antifungal lipopeptides—equips strain 10-4 with a powerful defensive and competitive toolkit, underpinning its potential for effective biocontrol and ecological success in the plant rhizosphere.

### 2.3. Growth-Simulating Impact of B. subtilis 10-4 on Plants Under Laboratory and Field Conditions

**Figure 5:**
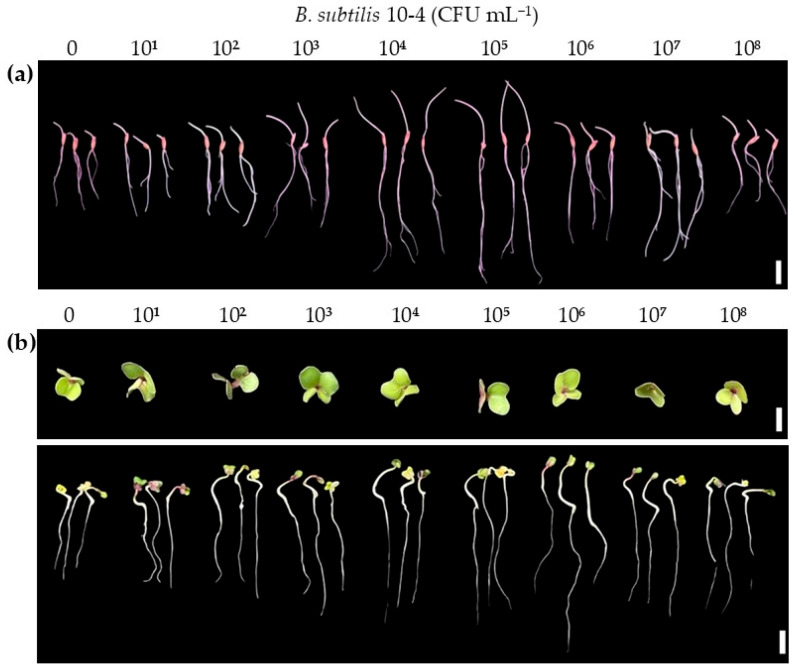
Representative images of 5-day-old oat (**a**) and radish (**b**) plants from laboratory experiments following pre-sowing seed inoculation with endophytic bacterium *Bacillus subtilis* 10-4 in different concentrations (0–10^8^ CFU mL^−1^). Scale bars: 0.5 cm.

To experimentally validate the PGP traits of strain 10-4 predicted from genomic analysis, we evaluated its effect on the seed germination of oat (a monocot) and radish (a dicot). The results presented in Table 5 and Figure 5 demonstrate the influence of *B. subtilis* 10-4 exhibited a clear dose-dependent effect with a distinct optimum for both crops. For radish, a statistically significant increase in germination was observed within the concentration range of 10^3^–10^7^ CFU mL^−1^, with peak effectiveness (a 136% increase relative to the control) achieved at 10^6^ CFU mL^−1^. At this optimum, inoculated radish seedlings exhibited more vigorous growth, characterized by longer and sturdier hypocotyls and the rapid emergence of well-expanded cotyledons compared to the control. In contrast, the highest tested concentration (10^8^ CFU mL^−1^) did not exert a significant effect compared to the control. A similar trend was observed for oat. The maximum stimulating effect, a 131% increase in germination, was observed at 10^5^ CFU mL^−1^. Seedlings at this concentration demonstrated accelerated coleoptile emergence and developed longer, more robust primary roots than the untreated control. Although the effect at 10^8^ CFU mL^−1^ remained higher compared to control, it was significantly lower than that achieved at the optimal concentration. Notably, inoculation with lower bacterial concentrations (10^1^ and 10^2^ CFU mL^−1^) did not induce significant effect on germination in either tested crop, suggesting a threshold level required for successful plant colonization and triggering growth stimulation mechanisms. Thus, the best stimulating effects for oat and radish were achieved at 10^5^ CFU mL^−1^ and 10^6^ CFU mL^−1^, respectively.

**Figure 6:**
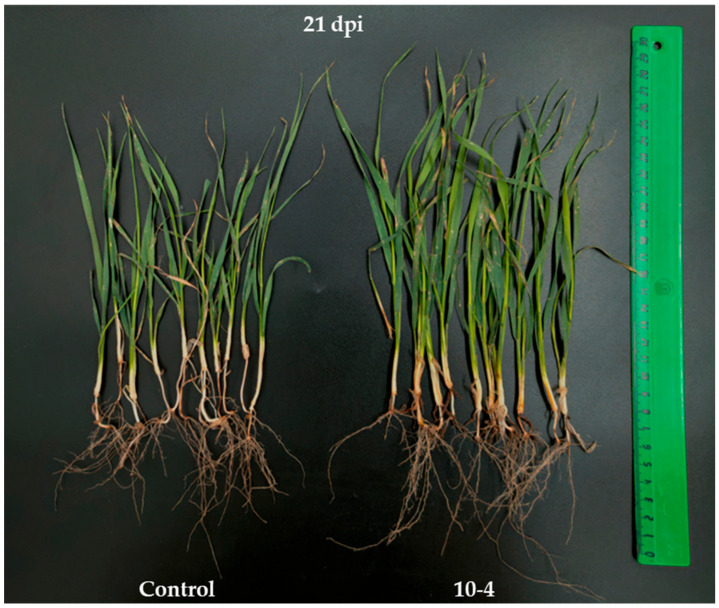
Representative images of wheat plants from field experiments following pre-sowing seed inoculation with endophytic bacterium *Bacillus subtilis* 10-4 (10^5^ CFU mL^−1^); dpi—days post inoculation.

Under field conditions, pre-sowing seed inoculation with *B. subtilis* 10-4 (10^5^ CFU mL^−1^) resulted in a significant increase in wheat growth parameters during the vegetation (Table 6, Figure 6). Particularly, bacterial inoculation increased in 21- and 54-day-old plants, respectively, the length of roots by 198% and 122%, the length of shoots by 109% and 102%; the roots’ fresh weight (FW) by 150% and 121%, shoots’ FW by 178% and 120%; roots’ dry weight (DW) by 163% and 167%; shoots’ DW by 166% and 126%. The results demonstrate the highest values in the length and FW of plants (roots and shoots), as well as shoots’ DW at early growth stages (21 dpi in comparison to 54 dpi), while the changes in roots’ DW were higher at the same level (163–167%) in comparison to control. Moreover, it was revealed that bacterial inoculation led to the formation of more productive stems per one wheat plant in comparison to control.

Thus, the results indicate that growth-promoting and antistress impacts of *B. subtilis* 10-4 on plants under laboratory and field conditions are associated with the presence in its genome of diverse genes responsible for colonization (adhesion, motility, chemotaxis), biosynthesis of amino acids and derivatives, carbohydrates, fatty acids, secondary metabolites with phytohormonal (IAA and SA), antimicrobial (proteases, glucanases, surfactin, bacillibactin, bacilysin, subtilosin A, plipastatin, terpenes, sactipeptide and ranthipeptide) activities, metabolism of nitrogen, potassium, phosphorus, sulfur, iron, VOCs, vitamins, proteins, tolerance to harsh conditions, cell signaling, dormancy and sporulation, respiration, cell wall and capsule, and general stress response regulation. Crucially, these in silico genomic predictions were functionally supported by a dose-dependent increase in seed germination efficiency in both monocot (oat) and dicot (radish) plants under laboratory conditions and wheat growth under field conditions; this underscores the importance of strain-specific inoculation protocols to maximize its beneficial effects.

## 3. Discussion

Building upon our previous findings, which demonstrated the ability of endophytic PGP bacterium *B. subtilis* 10-4 to enhance plant growth and stress tolerance [38,39,40,41,42,43,44,45,46,47,48,49,50,51,52,53,54], this study leveraged whole-genome sequencing to elucidate the genetic basis of these beneficial traits. The genomic data confirmed the strain’s membership in the *B. subtilis* species and revealed a robust repertoire of genes associated with plant growth promotion, aligning with the known capacity of this clade for plant colonization and interaction [15,16,17,18,19,20,21,22,23,24,25]. Specifically, our analysis identified in strain 10-4 a comprehensive set of genetic determinants for the synthesis of phytohormones like IAA [59,60,61,62,63,64], osmolytes [60,61,62], antimicrobial compounds for biocontrol [22,23,57,60,63,64], systems for nutrient acquisition, and inducers of systemic resistance [16,18].

Colonization is a primarily crucial factor for establishing close plant–microbe interactions and influencing biological activity of PGP bacteria and is a prerequisite for phytostimulation (growth promotion) and enhanced stress resistance in combination with other mechanisms (i.e., synthesis of osmolytes, hormones, and antioxidants) [60,61,62]. Earlier, using the surface-sterilized seedlings and random amplified polymorphic DNA (RAPD)-PCR, we showed that *B. subtilis* 10-4 successfully colonized the root surface and internal tissues (endophyte) of plants: wheat [41,42], bean [38], and potato [40]. In silico analysis predicted a set of genes involved in biofilm formation and plant colonization in the genome of *B. subtilis* 10-4 (Table 3) and provides a plausible genetic explanation for previously observed colonization of internal plant tissues by this bacterium [38,40,41,42]. It was also predicted a suite of genes in the *B. subtilis* 10-4 genome associated with plant growth and mineral nutrition. These include genes responsible for auxin production, nitrogen, phosphorus, potassium, and sulfur metabolism, as well as iron uptake and metabolism (Table 3). These genomic predictions align with the known phytostimulatory effects of other *Bacillus* species [33,61,62] and are directly corroborated by our prior in vitro experimental data, which confirmed that strain 10-4 produces IAA and siderophores along with its capacity for atmospheric nitrogen fixation—verified through combined phenotypic analysis (growth on nitrogen-free medium) and direct quantification via gas chromatography [38,41,51].

Among the PGP properties, auxins play a vital role in plant growth and development [65]. IAA is the most common auxin, and its precursor, tryptophan (Try), has been found in many PGP bacteria [66]. IAA regulates plant cell division, elongation, differentiation, seed germination, root development, and vegetative growth [65]. Both plants and bacteria have Try-dependent and Try-independent pathways, and bacteria may have multiple pathways simultaneously [66,67]. By today, Try-dependent IAA production in microorganisms via indole-3-acetamide (IAM), indole-3-pyruvate (IPA), tryptamine (TAM), indole-3-acetonitrile (IAN), and tryptophan side-chain oxidase (TSO) pathways was found [66]. These pathways do not always exist separately in a microorganism and may form interactive effects. In our study, in silico analysis of the *B. subtilis* 10-4 genome predicted the genetic potential for IAA biosynthesis via the Try-dependent pathway (Table 3). This genomic prediction was directly confirmed by our prior experimental data, which demonstrated the strain’s ability to synthesize IAA in the presence of Try [41]. Furthermore, the observation that IAA production persisted in the absence of Try [51] suggests the additional involvement of Try-independent pathways. The Try-independent pathway may play a functional role, potentially acting as a compensatory mechanism to increase IAA levels when the underlying Try-dependent pathways are impaired or less active [66]. This genetic architecture likely underpins the metabolic flexibility that enables sustained phytohormone production under varying environmental conditions. To fully elucidate the contribution of each pathway, future work employing targeted mutagenesis of specific pathway genes coupled with HPLC-MS quantification of IAA output under controlled conditions with and without Try precursors.

Improving the availability and digestibility of mineral nutrients from the soil for plants is another important mechanism of plant growth enhancement by PGP bacteria, which is achieved by converting indigestible forms into those that are digestible for plants. Our results showed the presence in *B. subtilis* 10-4 of complete gene clusters for siderophore production (Table 3), which facilitate high-affinity chelation of Fe^3+^. These siderophores support plant growth by enabling iron transport into plant cells, where it functions in essential processes including ATP synthesis, DNA precursor production, and heme formation [68]. Additionally, siderophore production provides a competitive advantage during plant colonization and pathogen exclusion. This genomic finding establishes the molecular basis for the siderophore-producing capability of strain 10-4 that we previously confirmed through experimental assays [41].

For phosphorus (P) metabolism, in the *B. subtilis* 10-4 genome, the encoding components of the high-affinity phosphate-specific transport (Pst) system and other phosphorus metabolism functions were predicted (Table 3). As is known, P plays crucial roles in carbon and energy metabolism, membrane formation, and synthesis of essential biomolecules, including ATP, nucleic acids, and phospholipids [69]. The Pst system enhances mineralization of recalcitrant soil organic P by upregulating microbial phosphatases, thereby increasing its bioavailability for plant uptake [69]. Particularly, in our study the genomic analysis identified in *B. subtilis* 10-4 a set of genes essential for P solubilization and transport, including *pstS* (phosphate-binding protein), *pstA*, *pstB*, *pstC* (phosphate transport system components), and *pitA* (low-affinity inorganic phosphate transporter). This genetic repertoire indicates robust capability for improving P bioavailability, which was functionally validated by our previous work demonstrating enhanced P accumulation in potato plants inoculated with this strain [40]. The same study [40] also revealed an increase in plant nitrogen content, suggesting a coordinated beneficial effect on plant nutrition. This finding aligns with established knowledge that bacterial endophytes can participate in multiple nitrogen cycle processes, including aerobic nitrification, microaerobic nitrogen fixation, and anaerobic denitrification, as genomic evidence from various microbial systems has reported [70]. Regarding nitrogen metabolism, the 10-4 genome contains a coordinated genetic system for nitrogen assimilation, including regulatory genes (*glnG*, *nrgB*, *glnG*, and *nrgB*) and enzymes for glutamate/glutamine metabolism (*gltA*, *gltB*, *glnA*, and *fpgS*). This genetic foundation supports the strain’s observed N_2_ fixation capability, which we previously validated through several approaches: direct measurement by gas chromatography, growth on Ashby nitrogen-free medium [39,41], and demonstrated enhancement of nitrogen accumulation in inoculation plants [40]. These coordinated systems likely enable efficient adaptation to varying nitrogen availability.

*Bacillus* spp. is well-known for producing numerous metabolites with biocontrol activities, including antimicrobial peptides (AMPs), both ribosomally (bacteriocins) and non-ribosomally synthesized (NRPS), as well as polyketides (PKs) that play a crucial role in innate immunity because they inhibit or kill a diverse range of pathogens, thereby indirectly enhancing plant growth [59,64,71]. For instance, Hanif et al. [72] reported that fengicin produced by *B. amyloliquefaciens* FSB 42 directly acts on the *Fusarium graminearum*’s cell membranes, causing an outflow of cellular contents and ultimately leading to the death of the pathogenic fungi. The antiSMASH analysis revealed a diverse repertoire of BGCs in *B. subtilis* 10-4. In comparison with the well-studied strains such as *B. subtilis* 168 and plant-beneficial *B. subtilis* Bbv57 [57], the genome of strain 10-4 is distinguished by a distinctive combination of variant lipopeptide clusters and a unique terpene biosynthetic pathway, suggesting the unique mode of action and significant biocontrol potential. Genome analysis predicted numerous specialized secondary metabolites with antimicrobial activity (e.g., bacillibactin, bacillaene, subtilosin A, surfactin, fengicin, plipastatin, and bacilysin) (Table S1), which likely provides a genetic basis for its experimentally demonstrated ability to suppress phytopathogens, including *Fusarium* spp., *Phytophthora infestans* [44,45,47], and *Alternaria alternata* [46]), as documented in our previous in vitro and in planta studies [44,45,46,47]. Furthermore, the strain possessed an extensive arsenal of antimicrobial lipopeptides, complementing its unique terpene pathway. Crucially, the genomic prediction of non-ribosomal peptide synthesis was empirically validated for the key antimicrobial compound surfactin. The genome of *B. subtilis* 10-4 harbors the complete srfAA–AD operon encoding surfactin synthase, and the production of surfactin (C13–C15 isoforms) by this strain was conclusively confirmed using the high-performance liquid chromatography-mass spectrometry (HPLC-MS) method in our previous work [47]. Beyond surfactin, the biocontrol potential of strain 10-4 is further supported by its genomic capacity to produce hydrolytic enzymes. We identified genes encoding proteases, β-glucanase (*bglS*), and endoglucanase (*eglS*), which are known to degrade the cell walls of pathogenic fungi and bacteria. A common mechanism of suppression in *Bacillus* spp. [69,73].

*Bacillus*-induced systemic tolerance represents a key mechanism for enhancing plants resilience to abiotic stressors (drought, salinity, temperature extremes) [13,15,31,36,37]. Our genomic analysis of *B. subtilis* 10-4 reveals a sophisticated genetic foundation for stress adaptation, featuring genes encoding critical stress-response proteins (Table 4). These include multiple superoxide dismutases (*sodA*, *sodB*, *sodC*, *sodMn*) for oxidative stress detoxification, a function crucial for bacterial survival in the rhizosphere, as demonstrated in other systems [74]. The strain 10-4 also possesses the *OpuD* gene for glycine betaine transport, which is known to confer a high degree of osmotolerance in *B. subtilis* [75], as well as *putP* (proline transporter), *hslR* (heat shock protein) genes, and the *speE* gene for polyamine spermidine biosynthesis. Notably, spermidine produced by *B. subtilis* OKB105 has been shown to enhance plant growth by modulating ethylene signaling and activating cell-expansion pathways [76], suggesting a potential conserved mechanism for strain 10-4. These genetic determinants correlate directly with our experimental observations of *B. subtilis* 10-4′ tolerance to drought (PEG-6000), herbicides [48], and cadmium toxicity [43]. Furthermore, they provide a molecular explanation for the previously demonstrated ability of the strain 10-4 to enhance plant tolerance to various stresses, including drought [42,51], salinity [38,39], cadmium toxicity [43], and even stress combinations (e.g., drought with *F. culmorum* [45] and herbicide [48]). The presence of specialized transport systems (*NhaA*, *NhaD*, and *nptA*) and regulatory transcription factors in the 10-4 genome (Figure 4) further supports its capacity for effective plant colonization and metabolic adaptation.

Many microorganisms are capable of emitting VOCs during metabolism [77]. *Bacillus*-emitted VOCs promote the growth of host plants and modulate defense responses [78,79]. *B. subtilis* 10-4 genome analysis revealed the genes encoding VOCs: *alsS* (acetolactate synthase), *alsD* (alpha-acetolactate decarboxylase), and *bdhA* (D-beta-hydroxybutyrate dehydrogenase), which enhance plant growth and systemic resistance [78], as well as the genes *gabD* (succinate-semialdehyde dehydrogenase [NADP(+)), *pdxT* and *pdxS* (pyridoxal 5′-phosphate synthase subunits PdxT and PdxS). The *gabD* gene in bacteria encodes an enzyme involved in the degradation of GABA, which plays an important role in the GABA shunt pathway, allowing certain bacteria to utilize GABA as a nitrogen source. The *PdxT* gene plays a role in the synthesis of pyridoxal 5′-phosphate in bacteria, which is the active form of vitamin B6 and is an important cofactor for many enzymes and reactions in bacteria [80]. The *pchA* gene found in the *B. subtilis* 10-4 genome indicates its potential to produce SA, which is an important metabolite that can be used by bacteria in a variety of biological processes, including defense mechanisms, signaling pathways, and environmental adaptation [81]. Since the *pchA* gene encodes an isochorismate synthase (ICS) enzyme, which is a key enzyme in SA biosynthesis. ICS utilizes isochorismate, an intermediate in the metabolic pathway, to synthesize SA [82]. Our previous studies demonstrated the significant role of SA in *B. subtilis* 10-4, which caused drought stress tolerance of wheat plants [52].

This genomic study provides robust in silico evidence that uncovers the genetic basis for the plant-beneficial effects of *B. subtilis* 10-4. The functional outcome of this genetic potential is supported by our previously observed PGP traits and new experimental phenotypic data from laboratory (radish, oat) and field (wheat) trials. The logical next step involves directly quantifying the predicted metabolites and evaluating the stress tolerance of inoculated plants under controlled and field conditions. Future research should focus on coupling transcriptomic analyses with chemical detection methods (e.g., HPLC-MS) to quantify the production of key bioactive compounds like fengycin, VOCs, GABA, and SA, and measure their impact on plants under different climate-change-associated stress combinations. Furthermore, targeted mutagenesis of specific genes would be essential to unequivocally establish their role in the observed PGP and resilience. This integrated approach will bridge the gap between genetic potential and mechanistic understanding, solidifying the strain’s value for agricultural application.

## 4. Materials and Methods

### 4.1. Bacterial Strain, Cultivation, and Inoculum Preparation

*B. subtilis* 10-4 is a PGP bacterium previously isolated from the arable layer of dryland soil at the Laboratory of Plant–Microbe Interaction of the Bashkir Research Institute of Agriculture, Ufa Federal Research Center of the Russian Academy of Sciences (UFRC RAS), identified using 16S rRNA and characterized on PGP traits [38,41], and deposited in the All-Russia Collection of Industrial Microorganisms as a promising agent for the development of bioinoculant formulations (VKPM, reg. no. B-12988). The colonies in Luria Bertani (LB) medium are round with a wavy edge, beige-white in color, and with a smooth surface. The cells are regular rods with rounded ends and monopolar peritrichous flagella. The cell size is 1.5–2.0 × 0.8–1.0 μm. Growth in liquid and semi-liquid nutrient media is microaerophilic, and metabolism is respiratory. *B. subtilis* 10-4 is capable of colonizing plant tissues (endophyte) and promoting their growth under normal and stress conditions (i.e., salinity [38,39,41], drought [42,51], Cd stress [43], and pathogens [44,45,46]).

*B. subtilis* 10-4 cells were cultured in the LB solid medium in Petri dishes (37 °C, 24 h) or in the liquid LB (37 °C, 180 rpm, 24 h). The cell concentration was determined at 600 nm (SmartSpecTM Plus, Bio-Rad, Hercules, CA, USA). The obtained bacterial culture was diluted with sterile water to the required concentrations for the experiments.

### 4.2. Genomic DNA Extraction, Whole Genome Sequencing, and Assembly

Whole-genome shotgun sequencing (WGS) of the *B. subtilis* strain 10-4 was performed on a Miseq/NextSeq automated sequencer (Illumina Inc., San Diego, CA, USA) at the Multi-Omics Technologies of Living Systems Research Laboratory of Kazan Federal University (Kazan, Russia). Bacterial DNA was isolated using the commercial FastDNA™ SPIN Kit for Soil (MP Biomedicals, Irvine, CA, USA). The quality of the isolated DNA was assessed by visualization in 0.8% agarose gel. Quantitative assessment was performed on a Qubit^®^ 2.0 fluorometer (Invitrogen, Carlsbad, CA, USA). Fragmentation of 1 µg of genomic DNA was performed by ultrasound using a Covaris S220 device (Covaris Inc., Woburn, MA, USA); the DNA library was prepared using NEBNext Ultra II kits (NEB, Ipswich, MA, USA) according to the manufacturers’ instructions. The quality of the resulting DNA fragment library was assessed on the chips of the 2100 Bioanalyzer instrument (Agilent Technologies, Santa Clara, CA, USA). Filtering of reads from adapter sequences was performed using the fastp v. 0.23.2 software (https://github.com/OpenGene/fastp (accessed on 21 July 2023)), de novo genome assembly was conducted with SPAdes v. 3.15.3 (https://github.com/ablab/spades (accessed on 21 July 2023)), and annotation was carried out using prokka v. 1.12 (https://github.com/tseemann/prokka (accessed on 21 July 2023)).

### 4.3. Genome Annotation and Phylogenetic Tree Construction

The circular genome was generated using the Proksee server (https://proksee.ca/ (accessed on 12 May 2025)) [83] and (BLAST Ring Image Generator (BRIG) software v. 0.95 [84]. The whole genome sequence data of *B. subtilis* 10-4 were uploaded to the Type (Strain) Genome Server (TYGS), a free bioinformatics platform (https://tygs.dsmz.de (accessed on 23 July 2025)) for a whole genome-based taxonomic analysis [85]. The analysis also made use of recently introduced methodological updates and features [86]. Information on nomenclature, synonymy, and associated taxonomic literature was provided by TYGS’s sister database, the List of Prokaryotic Names with Standing in Nomenclature (LPSN) (https://lpsn.dsmz.de (accessed on 23 July 2025)) [86]. For the phylogenomic inference, all pairwise comparisons among the set of genomes were conducted using Phylogenomic tree BLAST Distance Phylogeny (GBDP), and accurate intergenomic distances were inferred under the algorithm ‘trimming’ and distance formula *d5* [86]. 100 distance replicates were calculated each. Digital DDH values and confidence intervals were calculated using the recommended settings of the GGDC 4.0 [86,87]. The resulting intergenomic distances were used to infer a balanced minimum evolution tree with branch support via FASTME 2.1.6.1, including SPR postprocessing [88]. Branch support was inferred from 100 pseudo bootstrap replicates each. The trees were rooted at the midpoint [89] and visualized with PhyD3 [90]. The type-based species clustering using a 70% dDDH radius around each of the 12 type strains was performed as previously described [91]. Subspecies clustering was done using a 79% dDDH threshold as previously introduced [85].

### 4.4. Functional Annotation and Genomic Properties of Strain 10-4

Functional annotation of the bacterial genome was performed using the Rapid Annotation using Subsystem Technology v2.0 (RAST) web server (https://rast.nmpdr.org (accessed on 12 May 2025)). The assembled WGS of *B. subtilis* 10-4 is available at NCBI DDBJ/ENA/GenBank (Accession no. JAVHKX0000000000, Genome Project ID: PRJNA1008864) was submitted for analysis. The genes responsible for the production of secondary metabolites with antibiotic activities in the *B. subtilis* 10-4 genome were detected using the antiSMASH bacterial v.7.1./0 web server (https://antismash.secondarymetabolites.org (accessed on 27 April 2025)) [92,93].

### 4.5. Plant Growth Analysis

To confirm the repeatability of our previously obtained data on the beneficial effects of *B. subtilis* 10-4 on plants, additional experiments were conducted on its effect on other plant species under laboratory conditions and wheat plants under field conditions. In the experiments, as model plants, oat (*Avena sativa* L., Yakov), radish (*Raphanus sativus* L., Tambovchanka), and wheat (*Triticum aestivum* L., Ekada109) were used. For laboratory tests, the seeds of oat and radish (as representatives of monocotyledonous and dicotyledonous plants) were grown in Petri dishes (fifteen seeds/dish, three replicates) with 2 mL of bacterial suspensions in concentrations of 10^1^, 10^2^, 10^3^, 10^4^, 10^5^, 10^6^, 10^7^, and 10^8^ CFU mL^−1^ (tests) or dH_2_O (control) in the dark at 22 °C and 60% relative humidity (RH). After 5 days, the length of roots was measured [94].

Field experiments were carried out at the Experimental Farm of Bashkir Research Institute of Agriculture UFRC RAS (54°35′38″ N 55°23′42″ E, Chishmy, Russia) in 2025 on small plots (5 m^2^) (four replicates) [95]. Soil characteristics of the experimental field: leached chernozem with a heavy loamy mechanical composition, pH_KCl_—7.03, the contents of humus in the arable layer—7.4%, mobile potassium—140.0 mg kg^−1^ of soil, mobile phosphorus—105.14 mg kg^−1^ of soil, and nitrate nitrogen—6.90 mg kg^−1^ of soil. The soil was analyzed before sowing using standard methods [96,97,98,99]. The seeds were inoculated by soaking in *B. subtilis* 10-4 cell suspensions with concentrations of 10^5^ CFU mL^−1^ for 1 h. The suspensions were drained, and the seeds were air-dried. In control groups, the seeds were soaked in water. The inoculated seeds were sown into the soil at the time generally accepted for the region (the first half of May 2025). Plant growth parameters (length of roots, shoots, and biomass accumulation) were analyzed after 21 days (germination-tillering stage) and 54 days (earing stage) after sowing. The number of productive stems per plant was assessed in 54-day-old plants (earing stage). The length and weight of plants were measured using a ruler and scales, respectively. To determine the dry weight, plants (roots and shoots) were dried in an oven at 60 °C for 3 days (until a constant weight was achieved). In each group, 10 plants were analyzed in three replicates.

### 4.6. Statistical Analysis

All physiological experiments were carried out in three-four biological replicates. The results represented the average values of these replicates as the mean ± standard deviation (SD). The average data for 10 plants (analyzed in triplicate) are presented in Table 5. Prior to ANOVA, the assumptions of normality (Shapiro–Wilk test) and homogeneity of variances (Levene’s test) were verified and met (*p* > 0.05). Statistically significant differences between the mean values were determined using a one-way analysis of variance (ANOVA), followed by Tukey’s honesty significant difference (HSD) post hoc test (*p* < 0.05).

## 5. Conclusions

This study provides the first comprehensive genomic analysis of the endophytic PGP bacterium *B. subtilis* 10-4, whose growth-promoting and antistress effects on various plants have been well-established in our previous works. In silico functional annotation uncovered a sophisticated repertoire of genes conferring plant-beneficial traits, including: (i) direct plant growth promotion through phytohormone synthesis (e.g., auxins), nutrient solubilization (nitrogen, phosphorus, potassium), and siderophore production; (ii) biotic stress resistance via synthesis of diverse antimicrobials (e.g., bacilysin, bacillaene, bacillaene, subtilosin A, bacillibactin, surfactin, fengycin, plipastatin, and a unique terpene BGC with no known homologs) and other bioactive compounds; (iii) abiotic stress tolerance supported by genes for osmoprotectant synthesis (e.g., proline, betaine, GABA), oxidative stress detoxification (e.g., glutathione, SOD), and general stress response regulation; and (iv) host colonization efficiency facilitated by genes for chemotaxis, flagellar motility, biofilm formation, and EPS production. The functional relevance of this genetic potential was supported by the strains’ positive, dose-dependent efficacy in seed germination assays with oat and radish in the laboratory and, most importantly, by its ability to enhance wheat growth in the field. In general, this genetic insight establishes a mechanistic basis for the strain’s action and a genetic foundation for future targeted studies, such as transcriptomic analysis of plant–microbe interaction and the direct confirmation of predicted metabolites (e.g., via HPLC-MS), while also positioning it as a promising bioinoculant for enhancing crop productivity/resilience under changing climate.

## Figures and Tables

**Table 1:** *B. subtilis* 10-4 genome assembly and genome annotation data *.

Genome Size (bp)	4,278,582 (4.3 Mb)
Number of Contigs	19
Contig N50	496.9 kb
Contig L50	3
G+C Content (%)	43.5
Genome Coverage	99.0×
Assembly level	contig
Genes (total)	4476
CDSs (total)	4473
Genes (coding)	4314
CDSs (with protein)	4314
Genes (RNA)	53
rRNAs	1, 3, 2 (5S, 16S, 23S)
Complete rRNAs	1, 1 (5S, 16S)
Partial rRNAs	2, 2 (16S, 23S)
tRNAs	42
ncRNAs	5
Pseudo Genes (total)	109
Pseudo Genes (without protein)	109

* Assembly Method: SPAdes v. 3.15.3; Genome Representation: Full; Sequencing Technology: IonTorrent. Annotation Provider: NCBI; Annotation Pipeline: NCBI Prokaryotic Genome Annotation Pipeline (PGAP); Annotation Method: Best-placed reference protein set; GeneMarkS-2+; Annotation Software revision: 6.6.

**Table 2:** Comparative analysis of different *Bacillus subtilis* strains’ genomes.

Genome	Strains					
	*B. subtilis* 10-4	*B. subtilis* 26D	*B. subtilis* PTA-271	*B. subtilis* Bbv57	*B. subtilis* MBB3B9_DBT-NECAB	*B. subtilis* subsp. *subtilis* str. 168
NCBI number	PRJNA1008864	PRJNA1182114	RJNA646528	PRJNA794929	PRJNA786394	SAMEA3138188
Genome size (bp)	4,278,582	4,160,174	4,190,000	4,302,465	4,149,783	4,215,606
Genes (total)	4476	4342	4141	4363	4354	4135
CDSs (with protein)	4314	4133	3940	4281	4163	4120
rRNAs	1, 3, 2 (5S, 16S, 23S)	9, 7, 4 (5S, 16S, 23S)	9, 7, 4 (5S, 16S, 23S)	5	5, 1, 1 (5S, 16S, 23S)	30 (5S, 16S, 23S)
tRNAs	42	82	81	76	85	86
ncRNAs	5	5	5		5	1
Pseudo Genes (total)	109	102	95	27	94	13
Source	Soil	Cotton Leaves	Gravepine Rhizospheric Soil	Soil	Soil	Soil
Properties	Biocontrol, growth promotion	Biocontrol, growth promotion	Biocontrol	Biocontrol	Biocontrol, growth promotion	Reference Lab Domesticated

**Table 3:** Predicted genes associated with plant-growth-promotion (PGP) and mineral nutrition in *Bacillus subtilis* 10-4 genome.

PGP Activities	Gene Name	Function
Auxin biosynthesis	*trpA*, *trpB*, *trpC*, *trpP*, *trpD*, *trpE*, *trpF*, *TRPs*, *TSa*, *TSb*	Tryptophan biosynthesis
Nitrogen metabolism	*glnG*, *nrgB*	Regulation of nitrogen metabolism
	*NarG*, *NorD*, *NarH*, *NarJ*, *NorQ*, *NarI*	Denitrification
	*Amt*, *NsrR*	Assimilation
	*NarH*, *NarJ*, *NarG*, *NiR1b*, *NarI*, *NiR1a*	Ammonification of nitrates and nitrites
	*gltA*, *gltB*, *glnA*, *fpgS*	Glutamate/glutamine metabolism
	*narT*, *nasA*, *nasB*, *nasC*, *nasD*, *nasE*, *narX*, *narG*, *narH*	Nitrate/nitrite assimilation and metabolism
Phosphorus metabolism	*pstA*, *pstC*, *pstS*	Phosphate-binding and transport
	*pstB*	Phosphate transport system energetics
	*pitA*	Alternative phosphate transport
	*NaPi*, *PhoH*, *PhoP*, *PhoH*, *PpaX2*, *PhoR*	Phosphate metabolism
Iron acquisition and metabolism	*dhbC*, *FeuA*, *FeuB*, *dhbB*, *FeuC*, *dhbE*, *dhbF*, *yuiI*, *dhbA*, *Hyp1*, *Fe-ABC1*, *X-ABC3*, *X-ABC2*	Siderophores
	*X-ABC1*, *X-ABC3*, *HtsA*, *S*, *A*, *HtsB*, *ZnH*, *Hyp*, *HtsC*, *X-ABC2*, *R4*	Heme, hemin uptake and utilization systems in GramPositives
	*EfeB*, *EfeU*, *EfeO*	Ferrous iron transporter EfeUOB, low-pH-induced
Potassium metabolism	*KefA*	Potassium homeostasis
Sulfur metabolism	*AS*	Galactosylceramide and Sulfatide metabolism
	*Tpx*, *AhpC-like*, *TrxR*, *Bcp*	Thioredoxin-disulfide reductase

**Table 4:** Genes detected in genome of *Bacillus subtilis* 10-4 associated with stress response.

Trait	Gene Name	Function
General stress	*RsbWUSVRT*, *SigB*, *ysnF*, *yhdN* *CstA*, *Csr* *RseP*	Stress response regulation Carbon starvation Periplasmic stress
Drought, salt stress	*opuCA*, *opuCB*, *opuCC*, *opuCD*, *opuD*	Glycine betaine/choline transporter
Oxidative stresses	*gpx* *GloA* *sodA*, *sodB*, *sodC*, *sodMn*, *PerR,* *Osmcl*, *OsmclR*, *AhpC*, *NsrR*, *fur*	Glutathione: redox cycle Glutathione: non-redox reactions Protection against ROS Oxidative stress protection
Osmotic stress	*OpuD*, *OpuBA*, *GbsB*, *OpuAC*, *OpuAA*, *OpuBC*, *OpuBB*, *OpuBD*, *OpuAB*, *BetB* *glpF*	Choline and betaine uptake and betaine biosynthesis Osmoregulation

**Table 5:** Growth-stimulating impact of *Bacillus subtilis* 10-4 on the representatives of monocotyledonous (*Avena sativa* L.) and dicotyledonous (*Raphanus sativus* L.) plants in five days post inoculation (dpi) of seeds.

Parameter	*B. subtilis* Strain 10-4 Cells Concentration (CFU mL^−1^)								
	0 (H_2_O)	10^1^	10^2^	10^3^	10^4^	10^5^	10^6^	10^7^	10^8^
Oat (*Avena sativa* L.)									
Root Leight (cm)	1.6 ± 0.5	1.6 ± 0.6	1.7 ± 0.6	1.8 ± 0.6	2 ± 0.7	2.1 ± 0.6	1.9 ± 0.8	2 ± 0.6	1.8 ± 0.6
% of Control	100 ^e^	100 ^e^	106 ^d^	113 ^cd^	125 ^b^	131 ^a^	119 ^c^	125 ^b^	113 ^cd^
Radish (*Raphanus sativus* L.)									
Root Leight (cm)	2.1 ± 1.1	2.1 ± 1.3	2.1 ± 0.8	2.7 ± 1.3	2.8 ± 1.0	2.7 ± 1.4	2.9 ± 1.4	2.3 ± 1.0	2.1 ± 1.1
% of Control	100 ^e^	100 ^e^	100 ^f^	127 ^c^	127 ^c^	132 ^b^	136 ^a^	105 ^d^	100 ^e^

Lowcase letters indicate a significant difference between control and treatment groups (*p* < 0.05).

**Table 6:** Effect of seed inoculation with endophytic bacterium *Bacillus subtilis* 10-4 (10^5^ CFU mL^−1^) on the growth of wheat (*Triticum aestivum* L.) plants under field conditions in 2025 (dpi—days post inoculation).

Variant	Parameter												
	Length (cm)				Fresh Biomass (g)				Dry Biomass (g)				Number of Productive Stems Plant^−1^
	Roots		Shoots		Roots		Shoots		Roots		Shoots		
	dpi		dpi		dpi		dpi		dpi		dpi		dpi
	21	54	21	54	21	54	21	54	21	54	21	54	54
Control	6.4 ^bD^	11.0 ^bC^	34.7 ^bD^	79.4 ^bB^	0.12 ^bD^	2.93 ^aB^	1.97 ^bD^	25.0 ^bB^	0.09 ^bD^	2.33 ^bB^	0.90 ^bD^	11.06 ^bB^	1.0 ^b^
10-4	12.7 ^aB^	13.4 ^aA^	37.8 ^aC^	81.1 ^aA^	0.18 ^aC^	3.56 ^aA^	3.50 ^aC^	30.0 ^aA^	0.15 ^aC^	2.78 ^aA^	1.49 ^aC^	13.98 ^aA^	1.26 ^a^

Capital letters indicate a significant difference between all groups during experiment; Lowercase letters indicate a significant difference between control and treatment groups at one point (*p* < 0.05).

## Data Availability

The original contributions presented in this study are included in the article. Further inquiries can be directed to the corresponding author.

## Supplementary Materials

The following supporting information can be downloaded at: https://www.mdpi.com/article/10.3390/ijms262411904/s1.

## Abbreviations

The following abbreviations are used in this manuscript:

PGP	Plant Growth-Promoting
WGS	Whole-Genome Sequencing
BGCs	Biosynthetic Gene Clusters
SA	Salicylic Acid
IAA	Indole-3-Acetic Acid
LB	Luria–Bertani
CFU	Colony Forming Units
GABA	Gamma-Aminobutyric Acid
SOD	Superoxide Dismutase
ICS	Isochorismate Synthase
VOCs	Volatile Organic Compounds
EPS	Exopolysaccharide
