# Supplementary material for: Whole-Genome Analysis of PGP Endophytic Bacillus subtilis 10-4: Unraveling Molecular Insights into Plant Growth and Stress Resilience
Source: Int J Mol Sci. 2025 Dec 10;26(24):11904. doi: 10.3390/ijms262411904 (PMC12733254; doi:10.3390/ijms262411904)
Supplement: Supplementary file 1 [file ijms-26-11904-s001.zip › Supplementary Table S2.pdf]

Table S2. Identified regions of secondary metabolites in *Bacillus subtilis* 10-4 genome using anti-SMASH bacterial v.7.1.0

Region	Metabolite Type	Coordinates		Most similar known cluster	Similarity, %
		from	to		
1	Type III polyketide synthetase	858.006	899.103	1-carbapen-2-em-3-carboxylic acid	16
2	Non-ribosomal peptide synthetase, Betalactone	1	27.612	Fengycin	80
3	TransAT-polyketide synthetase, Type III polyketide synthetase, Polyketide synthetase -like	93.522	208.312	Bacillaene	100
4	Sactipeptide	412.249	433.860	Subtilosin A	100
5	Others	436.845	478.263	Bacilysin	100
6	Sactipeptide, Ranthipeptide	27.747	50.700	Sporulation killing factor	100
7	Non-ribosomal peptide synthetase	181.512	246.903	Surfactin	82
8	Non-ribosomal peptide metallophore, Non-ribosomal peptide synthetase	82.202	133.979	Bacillibactin	100
9	Terpene	67.005	88.903	-	-
10	Non-ribosomal peptide synthetase	1	14.356	Plipastatin, fengycin	23<br>20
11	Non-ribosomal peptide synthetase	1	9.446	Plipastatin, fengycin	15<br>13
12	Non-ribosomal peptide synthetase	1	7.967	Plipastatin, fengycin	46<br>20
